# Strengthening the Case for Universal Health Literacy: The Dispersion of Health Literacy Experiences Across a Southern U.S. State

**DOI:** 10.3928/24748307-20220620-01

**Published:** 2022-07

**Authors:** Iris Feinberg, Elizabeth L. Tighe, Michelle M. Ogrodnick

## Abstract

**Background:**

How individuals perceive their health literacy may differ based on demographic and individual characteristics.

**Objective:**

The purpose of this study was to understand the dispersion of health literacy across demographics in the state of Georgia in 2021 and to determine which factors influence health literacy.

**Methods:**

Study participants were age 18 years and older and completed an on-line Health Literacy Questionnaire (*N* = 520). The participant pool was stratified to mirror state-wide demographics of geography and race. Results were further collapsed into composite scales reflecting basic, communicative, and critical health literacy. Descriptive statistics, bivariate Pearson's correlations, and multiple regression analyses were used. A two-step cluster analysis was performed with the nine health literacy scales.

**Key Results:**

Rural county and no health insurance were negatively related to all three composite scales (*rs* = .093-.254, *ps* < .05). Demographic predictors accounted for 6.7% of the variance in basic (F[6, 439] = 5.287, *p* < .001), 10% in communicative (F[6, 438] = 8.154, *p* < .001), and 6% for critical (F[6, 439] = 4.675, *p* < .0010. In all scales, health insurance status was the strongest primary unique predictor (*βs* = .236, .295, .181, ps <.05, respectively). In a two-step cluster analysis only health insurance status differentiated the health literacy level clusters (*X*^2^(3) = 9.43, 34.51, *ps* = 024, <.001 respectively).

**Conclusion:**

Lacking health insurance is the most consistent and largest contributor to low health literacy across the state of Georgia; population demographics are not. Health literacy policies and practices should be developed for universal application and not focus on specific populations. [***HLRP: Health Literacy Research and Practice*. 2022;6(3):e182–e190.**]

**Plain Language Summary:**

In this study, demographics that are usually associated with low health literacy like age, sex, race, educational attainment, and type of county (rural or urban) were not associated with; the only significant factor was lack of health insurance. This relationship strengthens the case for universal health literacy precautions that go beyond population demographics.

The gap between the information patients, caregivers, and consumers find, understand, and use information to make health decisions and how that information is provided is immense and continues to widen. Health disparities are exacerbated by this gap; lack of health care access, low reading skills, health care costs, incomprehensible health guidelines, and a plethora of misinformation and disin-formation also contribute to inequitable health care and health outcomes. The unhealthiest states with high health disparities in the United States are in the Southeast including one-half of the states that have not increased access to health insurance through expansion of Medicaid under the Affordable Care Act. Our study was conducted in Georgia, which ranks in the bottom 10 states in terms of many health outcomes including low birth weight, chronic kidney disease, and sexually transmitted diseases. Factors other than health insurance also contribute to health inequities in Georgia; one-half of Georgia's 159 counties do not have enough primary care doctors to serve residents, 9 rural hospitals in Georgia have closed since 2008, 1 in 5 Georgians has low literacy skills, 11% of Georgians age 25 to 64 years do not have a high school diploma or equivalency, and Georgia ranks number 9 of 50 states in terms of income inequality between lowest and highest earning households (Sweeney, 2016; United States Census Bureau, 2021 b, c). Georgia ranks number 42 of 50 states regarding people who are uninsured and people with lack of health care access (America's Health Rankings, 2021; The Commonwealth Fund, 2020). These structural and individual factors increase health disparities.

The proliferation of information about the coronavirus disease 2019 (COVID-19) is a clear example of how challenges associated with an individual's low health literacy and structural inequalities can be exacerbated with devastating consequences (Gharpure et al., 2020; Roozenbeek et al., 2020; Spring, 2020). Poor COVID-19 knowledge and behaviors and poor health outcomes are associated with sociodemographic and structural characteristics that are also typically associated with low health literacy (HL) (i.e., race and ethnicity, sex, age, educational attainment, and low socioeconomic status) (Alsan et al., 2020; Karmakar et al., 2021). These characteristics are derived from HL studies that provide a single or range of summative scores and measure functional individual HL skills based on reading, numeracy abilities, and language comprehension (Nguyen et al., 2017).

HL encompasses both an individual's skills/abilities in receiving and using information and an organization/health providers' competencies in providing that information. For organizations, being health literate is more than just delivering health literate services and information; rather, the focus is on improving HL policies and practices throughout the organization (Brega et al., 2019). The Institute of Medicine Roundtable on HL published attributes and guidelines for organizations to help them create system-wide health literate organizations. There are 22 organizational-level quality improvement measures identified and assessed by HL experts that address each of the ten attributes called the Consensus Organizational Health Literacy Quality Improvement Measures; these measures can inform organizational HL initiatives (Brega et al., 2019). On the individual side, there are more than 100 available tools to measure individual HL skills; most focus on an individual's skill or ability to do certain tasks either within a certain disease state or general health care (U.S. Department of Health and Human Services, 2021). These tests measure varying domains of individual HL including conceptual knowledge, comprehension, numeracy, information seeking, appraisal and application of information, and speaker/listener communication. However, it is still unclear how an individual uses those skills when navigating the real-world of health care.

The clarion call for patient participation and shared decision-making can only be answered if people are able to fully engage in and navigate through the health care system. People need both knowledge and agency to participate; that is, they need to know what to do and have the skills and confidence to actually do it (McKenna et al., 2020). The elements in individual HL are complex and multifaceted; for example, finding health information requires, among other things, knowing what to look for, having access and skills to search for information, being able to read and understand what is written, and parsing through misinformation, disinformation, and conflicting information (Demner-Fushman et al., 2020). A more holistic approach also includes social determinants of health (SDOH), which are the structural conditions that affect health and health choices and include housing, education, income, access to health care, and transportation among others. Poor SDOH create barriers to finding, understanding, and using health information. In the broad definition of HL, finding information is a single construct, yet once a person has found information, there is little understanding of how either the process or the information itself improves HL or informs health decisions. Further, there is also little to connect organizational HL efforts to a patient's HL strengths and limitations.

The Health Literacy Questionnaire (HLQ) was developed to measure people's experiences of HL; that is, how they attempt to find, understand, and use health information and health services in the real world using cognitive and social skills (Osborne et al., 2013). The HLQ reports patient-reported outcome measures (PROMs) across nine scales, not direct measurement of skills or knowledge; data show that PROMs are relevant metrics for quality-of-life issues, organizational quality improvement, and better shared decision-making (Weldring & Smith, 2013). PROMs enable health care providers to understand what patients may find important and provide essential data to improve patient engagement. Each of the individual-level HLQ scales also provide valuable information for health organizations to consider when identifying interventions to improve both organizational and individual HL (Osborne et al., 2013).

One dominant theoretical approach in describing HL was developed by Nutbeam in 2001 and categorizes HL skills into basic communicative and critical literacy. According to Osborne et al., the HLQ's nine experiential domains can be organized into these three broad categories (2013). In some cases, the HLQ Domain Scales are in more than one level of Nutbeam's schema since some elements overlap. Understanding the specific HLQ Domain Scales through the wider perspective of the Nutbeam framework highlights how interventions designed for a specific domain can impact a broader set of HL skills. Further, Nutbeam's schema presumes progressive development along the HL spectrum as literacy, information, and cognitive skills develop in the realm of health and health care.

In this study, we used the HLQ measure to examine the HL experiences among individuals who live in a southern U.S. state. The HLQ measures are then organized according to Nutbeam's HL schema to create composite scales and better understand basic, interactive, and critical HL across the state. The aim of our study was to understand the dispersion of HL experiences across demographics typically associated with HL by answering the following research questions (RQ):

RQ1: What are the health literacy experiences of individuals across a southern U.S. state?

RQ2: Are there relations between demographics and basic communicative and critical health literacy composite scales?

RQ3: Are demographics predictive of the basic communicative and critical health literacy composite scales?

RQ4: Can we identify clusters of individuals with high and low health literacy based on the nine HLQ scales, and if so, how do those clusters differ by demographics?

## Methods

### Sample

Participants age 18 years and older who live in Georgia were recruited using Qualtrics Research Services (QRS). The participant pool was stratified to mirror state-wide demographics of geography and race (United States Census Bureau, 2010, 2021 a, d). Participants were sent an email invitation or prompted on the survey platform to proceed with the survey; interested respondents clicked on a hyperlink to access the survey. Participants were incentivized with cash, gift cards, or retail store miles according to their individual agreement with QRS. The study was approved by the Georgia State University Institutional Review Board.

### Measures

We collected demographic information on age, sex, race, highest level of educational attainment, health insurance status, and zip code. The HLQ measure was used to collect different aspects of HL experiences; the 44 questions generate nine scales with high reliability (overall Cronbach's alpha of >.08) (Osborne et al., 2013). Each of the nine scales contains 4 to 6 items scored on a Likert-style scale; scales 1 to 4 have four response options (*strongly disagree, disagree, agree, strongly agree*) and scales 5 to 9 have five response options (*cannot do, very difficult, quite difficult, easy, very easy*).

### Statistical Analysis

We used SPSS Version 27 for analysis. Descriptive statistics included means, standard deviations, frequencies, and chi-square calculations. We used bivariate Pearson's correlations as well as multiple regression analyses with the HL composite scales. A two-step cluster analysis was performed with the nine HL scales.

## Results

A total of 905 people accepted an invitation to the survey, with 520 meeting purposive stratification sampling criteria for geography (51% urban county, 49% rural county) and race (58% White, 32.1% Black or African American, 5.4% Asian, 2.5% Hispanic, 2% race and ethnicity not reported). These two sampling criteria matched overall state demographics. Respondents' age ranged from 18 to 80 years with an average of 36.3 years. Close to three-quarters were women. Approximately one-third of the participants were in each of three educational attainment categories (less than high school diploma, some college, college degree). Almost three-quarters of the participants had health insurance. All 520 fully completed the study with a 100% response rate. Demographic characteristics are shown in **Table 1**.

**Table 1:**
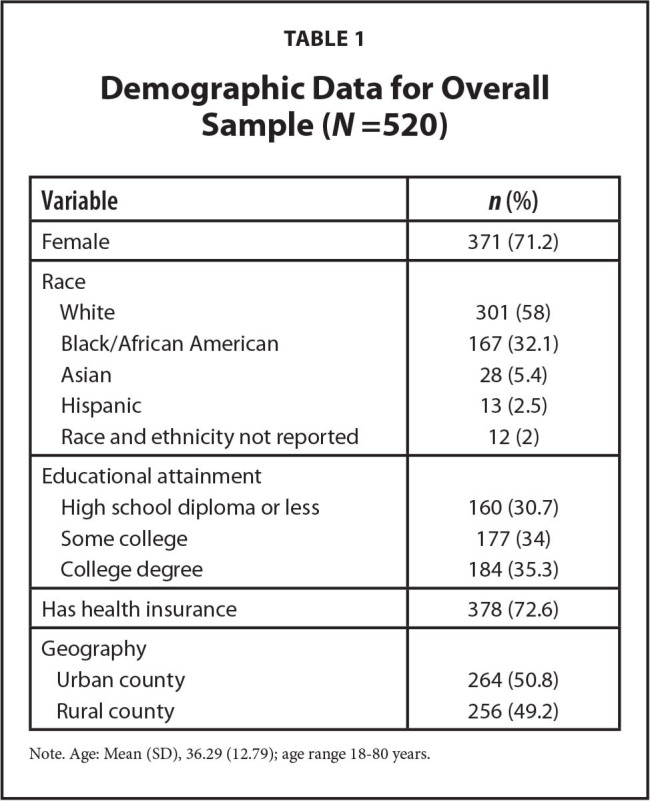
Demographic Data for Overall Sample (*N* =520)

**Variable**	***n* (%)**

Female	371 (71.2)

Race	
White	301 (58)
Black/African American	167 (32.1)
Asian	28 (5.4)
Hispanic	13 (2.5)
Race and ethnicity not reported	12 (2)

Educational attainment	
High school diploma or less	160 (30.7)
Some college	177 (34)
College degree	184 (35.3)

Has health insurance	378 (72.6)

Geography	
Urban county	264 (50.8)
Rural county	256 (49.2)

Note. Age: Mean (SD), 36.29 (12.79); age range 18–80 years.

To address RQ1, means, standard deviations, and minimum and maximum values on the nine HLQ scales are presented in **Table 2**. Scores for the first five scales ranged from 2.83 to 2.95 on a scale of 1 to 4. Scores for the next four scales ranged from 3.57 to 3.90 on a scale of 1 to 5.

**Table 2:**
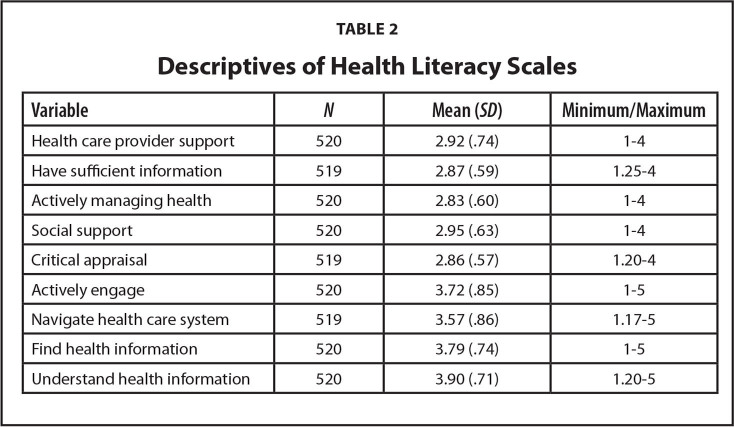
Descriptives of Health Literacy Scales

**Variable**	***N***	**Mean (*SD*)**	**Minimum/Maximum**
Health care provider support	520	2.92 (.74)	1–4
Have sufficient information	519	2.87 (.59)	1.25-4
Actively managing health	520	2.83 (.60)	1–4
Social support	520	2.95 (.63)	1–4
Critical appraisal	519	2.86 (.57)	1.20-4
Actively engage	520	3.72 (.85)	1–5
Navigate health care system	519	3.57 (.86)	1.17-5
Find health information	520	3.79 (.74)	1–5
Understand health information	520	3.90 (.71)	1.20-5

To address RQ2, three z-scored composite scales were created in alignment with Nutbeam's schema. Point-biserial and bivariate Pearson's correlations among the six demographic groups (age, sex, race, educational attainment level, county, and health insurance status) and three HL composite scales are reported in **Table 3**. As expected, the three composite scales were strongly and significantly and positively related (*rs* = .772−.904, ps <.001).

**Table 3:**
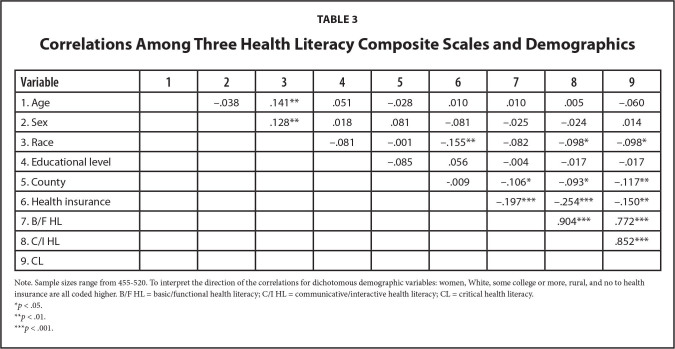
Correlations Among Three Health Literacy Composite Scales and Demographics

**Variable**	**1**	**2**	**3**	**4**	**5**	**6**	**7**	**8**	**9**
1. Age		−.038	.141^**^	.051	−.028	.010	.010	.005	−.060
2. Sex			.128^**^	.018	.081	−.081	−.025	−.024	.014
3. Race				−.081	−.001	−.155^**^	−.082	−.098^*^	−.098^*^
4. Educational level					−.085	.056	−.004	−.017	−.017
5. County						-.009	−.106^*^	−.093^*^	−.117^**^
6. Health insurance							−.197^***^	−.254^***^	−.150^**^
7. B/F HL								.904^***^	.772^***^
8. C/I HL									.852^***^
9. CL									

Note. Sample sizes range from 455-520. To interpret the direction of the correlations for dichotomous demographic variables: women, White, some college or more, rural, and no to health insurance are all coded higher. B/F HL = basic/functional health literacy; C/I HL = communicative/interactive health literacy; CL = critical health literacy.

* *p* < .05.

** *p* < .01.

*** *p* < .001.

To address RQ3, we ran three multiple regression analyses in SPSS Version 27. Each regression model contained the same six demographics as predictors and varied on the outcome (basic, communicative, and critical HL). For the first model (basic), the six predictors accounted for 6.7% of the variance (F[6, 439] = 5.287, *p* < .001; see **Table 4**). For the second model (communicative), the six predictors accounted for 10% of the variance (F[6, 438] = 8.154, *p* < .001; see **Table 4**). For the third model (critical), the six predictors accounted for 6% of the variance (F[6, 439] = 4.675, *p* < .001; see **Table 4**).

**Table 4:**
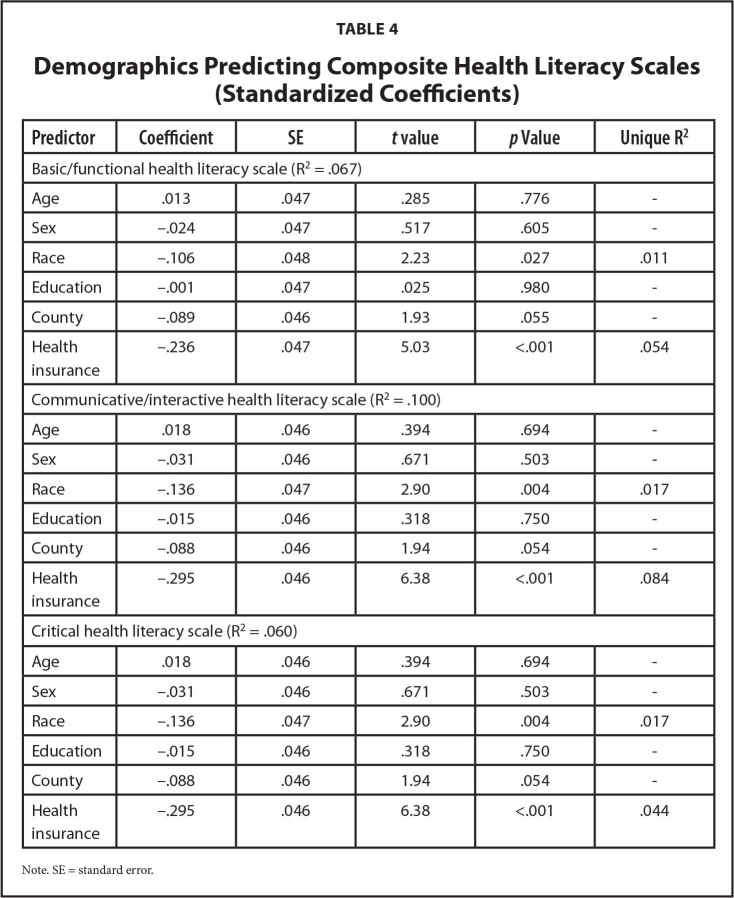
Demographics Predicting Composite Health Literacy Scales (Standardized Coefficients)

**Predictor**	**Coefficient**	**SE**	***t* value**	***p* Value**	**Unique R^2^**
Basic/functional health literacy scale (R^2^ = .067)					
Age	.013	.047	.285	.776	-
Sex	−.024	.047	.517	.605	-
Race	−.106	.048	2.23	.027	.011
Education	−.001	.047	.025	.980	-
County	−.089	.046	1.93	.055	-
Health insurance	−.236	.047	5.03	<.001	.054
Communicative/interactive health literacy scale (R^2^ = .100)					
Age	.018	.046	.394	.694	-
Sex	−.031	.046	.671	.503	-
Race	−.136	.047	2.90	.004	.017
Education	−.015	.046	.318	.750	-
County	−.088	.046	1.94	.054	-
Health insurance	−.295	.046	6.38	<.001	.084
Critical health literacy scale (R^2^ = .060)					
Age	.018	.046	.394	.694	-
Sex	−.031	.046	.671	.503	-
Race	−.136	.047	2.90	.004	.017
Education	−.015	.046	.318	.750	-
County	−.088	.046	1.94	.054	-
Health insurance	−.295	.046	6.38	<.001	.044

Note. SE = standard error.

**Figure 1.**
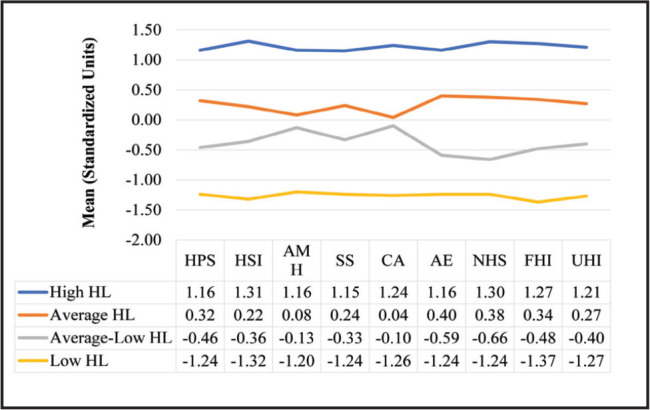
Four clusters based on nine health literacy scales. These are z-scored means on each of the nine scales. A positive value is indicative of higher responses on the nine health literacy scales (aligned closer with *strongly agree* or *agree* and *very easy *or *easy*). A negative value is indicative of lower responses on the nine health literacy scales (aligned closer with *strongly disagree* or *disagree* and *very hard* or *hard*). AE = actively engage; AMH = actively managing health; CA = critical appraisal; FHI = find health information; HL = health literacy; HPS = health care provider support; HSI = have sufficient information; NHS = navigate health care system; SS = social support; UHI = understand health information.

**Figure 2.**
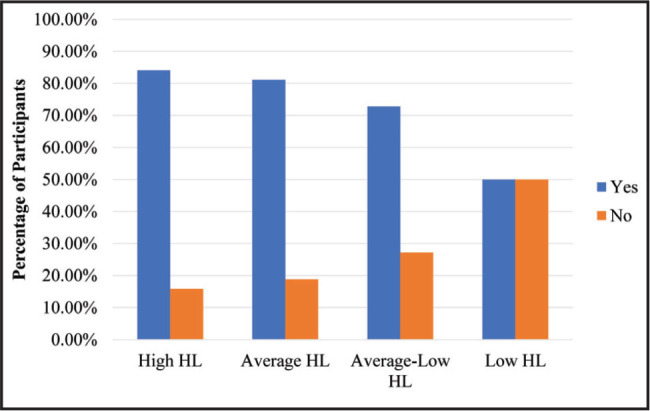
Percentage of participants by health insurance status and by clusters note. A chi-square test revealed significant differences among the clusters by county (X^2^[3] = 34.508, *p* < .001). Specifically, individuals without health insurance were more prevalent in the low health literacy (HL) cluster compared to the other three clusters.

To address RQ4, we ran a two-step cluster analysis in SPSS Version 27 with the nine HL scales. These scales were standardized (z-scored) prior to entering them into the cluster analysis. A two-step cluster analysis was preferred because this approach empirically determined the optimal number of clusters based on different combinations of our nine scales and we did not have an a priori number of clusters in mind. The results indicated that there were 4 distinct HL clusters based on the 9 scales (**Figure 1**). We have descriptively labeled the clusters as: *High HL* (17.2%, *n* = 89), *Average HL* (39.8%, *n* = 206), *Average-Low HL* (26.9%, *n* = 139), and *Low HL* (16.1%, *n* = 83). Across the nine scales, each cluster was fairly stable and homogenous in their responses. Only health insurance status significantly differentiated the clusters (*X*^2^(3) = 34.51, *p* < .05) (**Figure 2**).

## Discussion

This study finds that both race and lacking health insurance appear to be the most consistent contributor to HL across the state of Georgia. Demographic factors that are typically associated with HL show little association with HL levels, strengthening the case for universal HL precautions for all residents regardless of age, sex, educational attainment, or where they live. Race was related to 2 of the 3 HL composite scales (communicative and critical); however, it was not a differentiating factor between HL clusters; that is, there was no significant difference due to race in clusters of people who had higher or lower HL. In considering those HL levels, we find that having health insurance is the only differentiating factor among the demographics we studied. People who have health insurance may participate more in preventive and primary care; that is, they may have a regular health care provider, get annual health care checkups, receive health information from insurance companies, or be more confident navigating the health care system, all of which can lead to higher HL. Regular interactions with the health care system writ large can improve HL skills (Benjamin, 2010).

Age and sex have been shown to influence a person's HL level; these findings are based on numerous samples from test-based measurement studies. Individuals age 65 years and older on average have lower HL compared to younger adults (Centers for Disease Control and Prevention, 2021a; Kutner et al., 2006). Furthermore, in the National Assessment of Adult Literacy (NAAL), the percentage of adults age 65 years and older who had intermediate and proficient HL was lower when compared to younger adults (Kutner et al., 2006). Approximately 71% of people older than age 60 years had difficulty using print materials, 80% had challenges using forms or charts, and 68% struggled to interpret or do mathematical calculations (Kutner et al., 2006). Sex has also been shown to effect HL; generally, women tend to have higher HL compared to men (Clouston et al., 2017; Kutner et al., 2006). Our study showed, however, that when individuals self-report HL experiences in an online study, age and sex are not significant factors in HL in Georgia.

Racial and ethnic minority groups often have lower health literacy than White individuals. According to the NAAL, White and Asian/Pacific Islander adults on average had higher HL than adults who identified as Black, Hispanic, American Indian/Alaska Native, and multiracial (Kutner, et al., 2006). In a more recent study examining racial disparities in HL among patients with heart failure, African Americans/Black individuals were significantly more likely than White individuals to have poor HL (Chaudhry et al., 2011). Among other reasons, the difference in HL among African Americans/Black individuals and other underrep-resented racial and ethnic minorities stems from a history of discriminatory policies and practices such as limited access to resources, limited educational opportunities, racism, mistrust in the health care system, and lack of culturally appropriate health information (Muvuka et al., 2020). Our study participants matched the state's racial percentages [58% White, 42% BIPOC (Black, Indigenous, People of Color)]; however, we found that race had some influence on communicative or critical HL, but no effect on overall HL levels.

Through education, individuals develop cognitive function and reading ability (Lövdén et al., 2020). Research has shown a positive relationship between education and HL, meaning as educational attainment increases, so does HL (Muvuka, 2020; van der Heide et al., 2013). The NAAL reported that 49% of adults who did not complete high school had below basic HL. In comparison, 15% who had a high school diploma had below basic HL, and only 3% who had a bachelor's degree had below basic HL (Kutner et al., 2006). However, our study found that educational attainment in Georgia was not a factor relating to an individual's HL level.

Where a person lives can affect health and HL. Individuals who live in rural counties tend to have lower HL than those who live in urban areas (Chen et al., 2019; Golboni et al., 2018; Zahnd et al., 2009). However, individuals who live in rural compared to urban areas may face additional barriers that can influence these results. Rural county residents may face barriers like lack of hospitals, primary care and specialized physicians, and transportation that make it more challenging to access health information, and transportation (Aljassim et al., 2020; Chen et al., 2019). Residents of rural counties are more likely to have poor health, chronic diseases, and insufficient access to health care than those living in urban counties; these health disparities are often coupled with poor social determinants of health such as low-income level, low educational attainment, poor access to adequate housing, and food insecurity (Coughlin et al., 2019). Research shows that these factors should contribute to a lower HL level; however, this was not the case across Georgia.

According to the NAAL, individuals with health insurance through their employer, the military, or who privately purchased insurance on average had higher HL compared to individuals enrolled in Medicare, Medicaid, or who had no insurance (Kutner et al., 2006). Having health insurance enables individuals to access medical services when needed, including preventive care like an annual doctor's visit. Increased access to services increases the likelihood an individual will engage with the health care system. Being able to access and navigate the healthcare system takes time and practice. Therefore, it is not surprising that individuals who do not have experience using health insurance are unfamiliar with terminology and the process of getting care (Levitt, 2015). An individual's experiences with healthcare can influence current health practices, use of services, and sources of health information (Wolf et al., 2009). Therefore, having health insurance and access to care is crucial for improving health outcomes and HL and, in our study, is the only demographic factor correlated with HL levels.

Individuals without health insurance have more health disadvantages than those with health insurance such as poor health outcomes and high rates of mortality and premature death (America's Health Rankings, 2021). Inadequate access to quality care leads to undiagnosed or untreated chronic conditions, more emergency department visits, poor up-take of preventive services such as flu vaccines, expensive medical bills. The policy decision in Georgia to not expand Medicaid has a detrimental effect on the 1.4 million uninsured people in the state. Across all demographics, Georgia residents show a high prevalence of avoiding health care due to cost (America's Health Rankings, 2021). With Medicaid expansion, over 560,000 Georgians would be able to have health insurance (Cover Georgia, 2021). Closing this coverage gap yields both individual benefits and economic benefits including reductions in uncompensated care and financially strengthening struggling hospitals who are vulnerable to closure. Comparing states with similar demographic and economic factors, one study showed a 6% decline in all-cause mortality among adults age 20 to 64 years (Sommers, 2017). Other studies show states that broadly offer health insurance have better birth weight and diabetes prevalence outcomes (America's Health Rankings, 2021). Our study shows that having health insurance in Georgia is correlated with having higher HL which may translate into less health risk, more appropriate use of health system resources, positive self-care management, lower health costs, better understanding of health information, and higher levels of patient engagement in health decisions (Egbert & Nanna, 2009). Improving access to health insurance has been shown to reduce systemic disparities and health inequities. Expansion of health care through the Affordable Care Act has been shown to improve the share of insured Black, Hispanic, and White adults, decrease the share of adults who went without care because of cost, and increase the share of adults with a usual source of care rather than emergency department care (Baumgarten et al., 2020).

The health care system in the U.S. is complicated and takes considerable effort to navigate. Organizational HL refers to the steps that healthcare organizations can take to better serve their community members by providing understandable information and helping patients of various HL levels navigate the health care systems and manage their health (Brega et al., 2019). The ten attributes include making HL part of an organization's mission, integrating HL into planning and quality improvement, preparing employees to use health literate strategies, and providing accessible information to patients (Brach et al., 2012). When healthcare systems implement organizational HL, it can help patients navigate the system and be more engaged in managing their health (Agency for Healthcare Research and Quality, 2019). The attributes of organizational HL relate to the nine HLQ scale dimensions and the three HL composite scales with a focus on patient-centered care and ensuring that the patient has the tools and information necessary to participate in health-related decisions. In addition to revealing a lack of health insurance as a factor in individual HL, our study also highlights the need for organizations to implement universal HL guidelines and practices, regardless of race, sex, age, educational attainment, or type of county residence.

## Study Limitations

This study has several limitations. Data were collected through self-report, which can create social desirability bias in the responses and may affect validity of the responses. The validity of self-report scales may also vary by gender. The significant relationship between demographic factors typically associated with HL and the HL composite scales were only 6% to 10%; other demographic and SDOH factors could have been considered and may show different results. We only collected data from those who were able to access an on-line survey, which means that those with low digital access and/or literacy may have been missed. Although the gap in those with no insurance based on gender has closed dramatically since implementation of the Affordable Care Act, 2018 census data show there is still a gender gap: men are uninsured at a rate of 12.1% and women at 9.9% (Centers for Disease Control and Prevention, 2021b). Finally, prior data collected on the influence of demographics on HL were gathered using test-based measures; we report data from online self-report samples.
